# DAILY EFFECTS OF PERCEIVED AND ANTICIPATED STRESS ON SUBJECTIVE SLEEP QUALITY

**DOI:** 10.1093/geroni/igz038.2985

**Published:** 2019-11-08

**Authors:** Chelsea N Dickens, Martin J Sliwinski

**Affiliations:** 1 Pennsylvania State University, University Park, Pennsylvania, United States; 2 Pennsylvania State University, Center for Healthy Aging, University Park, Pennsylvania, United States

## Abstract

Prior studies of stress and sleep have postulated that anticipation of future stress may have a greater impact on sleep quality than stress experienced during the day. We examined this possibility using data collected over 14 consecutive days. Participants (Npersons=257, age=25-65) rated each evening how stressful the day was and how stressful they expected the next day would be. Each morning, participants rated their subjective sleep quality. After adjusting for the effect of the previous night’s sleep, on days when individuals reported feeling more stressed, they also reported not sleeping as well that night. However, expectations of stress the next day did not have an effect on sleep quality, suggesting that stress experienced during the day impacts sleep quality more so than anticipation of future stress. Despite previous findings that older age is associated with sleep complaints, age did not act as a moderator in our analysis.

